# Curbing action potential generation or ATP-synthase leads to a decrease in in-cell pyruvate dehydrogenase activity in rat cerebrum slices

**DOI:** 10.1038/s41598-021-89534-4

**Published:** 2021-05-13

**Authors:** Benjamin Grieb, Sivaranjan Uppala, Gal Sapir, David Shaul, J. Moshe Gomori, Rachel Katz-Brull

**Affiliations:** 1 Department of Radiology, Hadassah Medical Center, The Faculty of Medicine, Hebrew University of Jerusalem, 9112001 Jerusalem, Israel; 2 Department of Psychiatry and Psychotherapie I (Weissenau), Ulm University, ZfP Suedwuerttemberg, Ravensburg, Germany; 3 The Wohl Institute for Translational Medicine, Jerusalem, Israel

**Keywords:** Magnetic resonance imaging, Predictive markers, Neurological models

## Abstract

Direct and real-time monitoring of cerebral metabolism exploiting the drastic increase in sensitivity of hyperpolarized ^13^C-labeled metabolites holds the potential to report on neural activity via in-cell metabolic indicators. Here, we followed the metabolic consequences of curbing action potential generation and ATP-synthase in rat cerebrum slices, induced by tetrodotoxin and oligomycin, respectively. The results suggest that pyruvate dehydrogenase (PDH) activity in the cerebrum is 4.4-fold higher when neuronal firing is unperturbed. The PDH activity was 7.4-fold reduced in the presence of oligomycin, and served as a pharmacological control for testing the ability to determine changes to PDH activity in viable cerebrum slices. These findings may open a path towards utilization of PDH activity, observed by magnetic resonance of hyperpolarized ^13^C-labeled pyruvate, as a reporter of neural activity.

## Introduction

Recent advances in brain imaging have suggested that changes in brain energy metabolism could be monitored rapidly. Wehrl et al*.* have demonstrated a quick metabolic response in vivo*,* in a preclinical positron emission tomography (PET) study, which showed that whisker barrel stimulation resulted in increased ^18^FDG uptake^1^. A recent study by Grist et al*.* showed, by magnetic resonance (MR), rapid conversions of hyperpolarized [1-^13^C]pyruvate into both [1-^13^C]lactate and [^13^C]bicarbonate in the brains of healthy volunteers^2^. In the latter study, the sensitivity of detecting carbon-13 labeled metabolites was increased through hyperpolarization by a few orders of magnitude using the dissolution dynamic nuclear polarization (dDNP) technology^3^. In recent years, dDNP has been used to drastically improve the temporal and spatial resolution in comparison to thermal equilibrium nuclear magnetic resonance (NMR) spectroscopy in multiple preparations^4–8^. Hyperpolarized [1-^13^C]pyruvate is the benchmark metabolite used across different experimental dDNP designs including pioneering clinical studies on brain cancer imaging^9–11^. Preclinical studies showed a persistence of the hyperpolarized state in both [1-^13^C]lactate and [^13^C]bicarbonate in the brain of anesthetized rodents^12–15^, and we have previously shown that cerebral metabolism can also be measured using hyperpolarized [1-^13^C]pyruvate ex vivo*—*in perfused cerebral slices^4^. Thermal equilibrium NMR studies in rodent brain slices have shown utility in investigating brain metabolism over the last three decades^16–19^. In this setup ^31^P NMR has been used monitor the viability of slices by the presence of phosphocreatine and ATP signals^16,17^.

Using hyperpolarized [1-^13^C]pyruvate, the activities of both lactate dehydrogenase (LDH) and pyruvate dehydrogenase (PDH) can be monitored via the production of hyperpolarized [1-^13^C]lactate and [^13^C]bicarbonate, respectively. Using a recently developed acquisition approach termed product selective saturating-excitations, we have recently shown in perfused tissue slices that the activities of these enzymes can be quantified at a few seconds resolution and that instantaneous changes in apparent reaction rates can be determined without the use of kinetic modelling^8^.

Here, we applied the product selective saturating-excitations approach in a study of cerebral slices to investigate whether changes in neural activity will be reflected in the metabolic activities of hyperpolarized [1-^13^C]pyruvate utilization. To this end, we incubated cerebral slices with the canonical voltage-gated sodium-channel blocker tetrodotoxin (TTX) which is known to either diminish or altogether block action potential generation at concentrations of 0.2–10 μM^20–23^. Action potentials of mammalian neurons use sodium influx and potassium efflux causing a transient change in the resting membrane potential to encode and transmit information. The ionic gradient changes of this process are restored by the Na^+^/K^+^-ATPase pump, requiring metabolic activity due to the utilization of ATP^24^. Early studies on neuronal energy metabolism concluded that “the major energy-consuming function of activated nervous tissue is ion pumping of the sodium pump”^19^. Modeling of the energy budget for signaling in the grey matter of rodent brains predicted that the largest proportion of energy (47%) is consumed by action potentials, followed by postsynaptic glutamate signaling (34%)^25^. Furthermore, ^13^C-NMR spectroscopy revealed that both excitatory glutamatergic and inhibitory GABAergic neurotransmitter cycling and energy consumption scaled with neuronal activity^26^. Reverse microdialysis studies on rodent brains showed that local TTX infusion abolished firing activity of 40–60% of all monitored cells within a tissue sphere of 2 mm^22^. Specifically, incubation with 1 μM of TTX blocked the Na^+^ current in 300–400 µm rat brain slices^23^. Here we incubated 350 µm rat cerebral slices with 1 µM TTX to block action potential generation and thus reduce energy demand significantly. As a control, we have used cerebral slices that have not experienced any insult in the form of a pharmacological challenge. In addition, the TTX effect was also compared the effect of the antibiotic oligomycin (OLI^27^), which is an inhibitor of ATP-synthase.

## Materials and methods

### *Preparation of* cerebral *slices*

**Figure 1:**
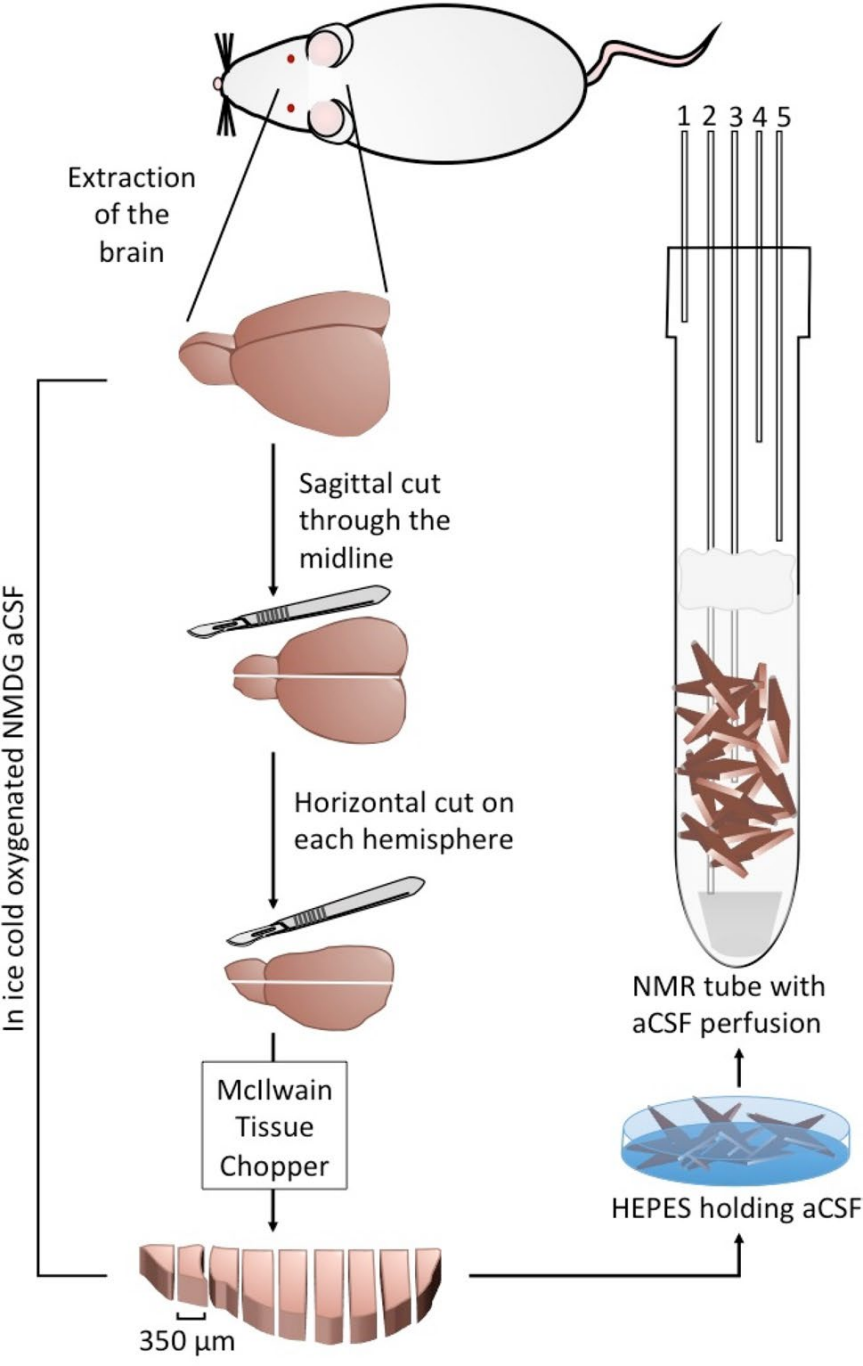
Schematic drawing of the surgery and the cerebrum slicing procedure. Following brain extraction, separation of the cerebellum, slicing of the cerebrum, and recovery period in HEPES-holding aCSF, the cerebral slices were placed in a 10 mm NMR tube with continuous perfusion of aCSF (Solution 3 below) and bubbled with 95%/5% O_2_/CO_2_ at 37 °C at a rate of 4 L/min. The lines going in and out of the NMR tube are marked as follows: 1. Humidified 95%/5% O_2_/CO_2_ atmosphere; 2. aCSF inflow line; 3. NMR compatible temperature probe; 4. Backup aCSF outflow line; 5. aCSF out-flow line. A filter made of cotton balls (colored gray in the Figure) was placed a few cm above the slices to prevent suction of the occasional floating slice into the out-flow lines. The slices were kept at the bottom of the tube at the region visible to the NMR probe due to gravity and possible inter-slice adhesion and the gentle perfusion generally did not displace them. A spacer made of solid PEEK was placed at the bottom of the tube prior to placing the slices in the NMR tube. This was done to raise the slices to the level observed by the NMR probe without the introduction of susceptibility differences at the region of the probe, thereby optimizing the use of cerebral tissue in each experiment.

The joint ethics committee (IACUC) of the Hebrew University and Hadassah Medical Center approved the study protocol for animal welfare (Ethical Protocol No. MD-16-14739-1). The Hebrew University is an AAALAC International accredited institute. The study was carried out in compliance with the ARRIVE guidelines^28^. The authors confirm that all experiments were performed in accordance with relevant guidelines and regulations. Female Sprague–Dawley rats (n = 7, 3–5 months old, 132–160 g) were obtained from the Hebrew University Authority of Biological and Biomedical Models. Care was taken to minimize pain and discomfort to the animals. Animals were housed in the animal facilities 3–5 days after delivery for acclimatization and fed ad libitum. On the experimental day, animals were transferred to the lab and anesthetized within one hour of arrival. After induction of deep anesthesia using isoflurane, transcardial perfusion, decapitation, and rapid brain removal were performed. The cerebellum was separated from the cerebrum and only the cerebrum was used in the current study. Slicing on a McIlwain tissue slicer to 350 μm thickness followed, and the obtained cerebral slices were separated into two batches and stored in oxygenated (95/5% O_2_/CO_2_) artificial cerebrospinal fluid (aCSF) at room temperature. This process is demonstrated in Fig. 1, which was modified from Harris et al.^4^.

### Chemicals

N-methyl-D-glucamine (NMDG), oligomycin from Streptomyces diastatochromogenes, and all other chemicals for solutions were obtained from Sigma-Aldrich (Rehovot, Israel). Tetrodotoxin was obtained from Alomone labs (Jerusalem, Israel). The OX063 radical (GE Healthcare, UK) was obtained from Oxford Instruments Molecular Biotools (Oxford, UK). [1-^13^C]pyruvic acid was purchased from Cambridge Isotope Laboratories (Tewksbury, MA, USA). The anesthetic isoflurane was obtained from the Hebrew University Authority for Biological and Biomedical Models.

### Solutions

To improve the health of acute cerebrum slices obtained from mature adult rats we modified a slice preparation technique optimized for in vitro electrophysiology in mature adult rat brain slices^29,30^. Ting et al*.*^29^ showed that the presence of NMDG during a 12 min recovery phase after slicing dramatically improved the health of acute slices from mature adult rats in electrophysiological recordings. NMDG is used as an extracellular replacer of sodium ions to prevent sodium ion and water influx into cells as the main insult causing cell death following brain slicing. Furthermore, incubation in HEPES-buffered artificial cerebrospinal fluid (aCSF) resulted in improved slice health^29^. Here, altogether six solutions were prepared for each experiment:

#### Solution 1

The NMDG-aCSF used for the brain perfusion procedure, tissue slicing, and slice recovery contained 93 mM NMDG, 2.5 mM KCl, 1.2 mM NaH_2_PO_4_, 26 mM NaHCO_3_, 20 mM HEPES, 25 mM D-glucose, 10 mM MgCl_2_, 0.5 mM CaCl_2_, 5 mM ascorbic acid, 2 mM thiourea, and 3 mM pyruvic acid in double distilled water.

#### Solution 2

The HEPES-holding aCSF contained 84 mM NaCl, 2.5 mM KCl, 1.2 mM NaH_2_PO_4_, 30 mM NaHCO_3_, 20 mM HEPES, 25 mM D-glucose, 2 mM MgCl_2_, 2 mM CaCl_2_, 5 mM ascorbic acid, 2 mM thiourea, and 3 mM pyruvic acid in double distilled water.

#### Solution 3

The aCSF used for perfusion in the NMR spectrometer contained 115 mM NaCl, 2.5 mM KCl, 1.2 mM NaH_2_PO_4_, 24 mM NaHCO_3_, 5 mM HEPES, 10 mM D-glucose, 2 mM MgCl_2_, and 2 mM CaCl_2_ in double distilled water containing 10% D_2_O.

All three solutions (1–3) were bubbled with 95%/5% O_2_/CO_2_ for at least 1 h prior to use and titrated to a pH of 7.2–7.4 using HCl and/or NaOH. All concentrations were optimized to result in a calculated osmolarity of 310 ± 15 mOsm.

#### Solution 4

The dissolution buffer contained 77 mM NaCl, 2.5 mM KCl, 15.7 mM TRIS, and 50 mM phosphate buffer and was titrated to reach a pH of 7.2–7.4 upon addition of 43 mM pyruvic acid.

#### Solution 5

A high solvent solution (6 × concentrated, *i.e.* 15 mM KCl, 7.2 mM NaH_2_PO_4_, 60 mM D-glucose, 12 mM MgCl_2_, and 12 mM CaCl_2_) was added to the dissolution buffer after dissolution to bring these solute concentrations in the hyperpolarized medium administered to the slices as close as possible to their concentration in Solution 3.

#### Solution 6

6 mL of Solution 4 mixed with 2 mL of Solution 5. The solution was placed in a conical tube at the fringe field of the magnet and was bubbled with 95%/5% O_2_/CO_2_ and pre-warmed in a 40 °C water bath.

Solution 6 together with the 4 mL of Solution 4 containing the hyperpolarized [1-^13^C]pyruvate make the hyperpolarized medium that was administered to the cerebrum slices.

### Surgery and slice handling

The procedure for obtaining rat cerebrum slices and handling them is depicted schematically in Fig. 1. We induced gaseous anesthesia with 3.5% isoflurane at a flow rate of 440 mL/min using a gas anesthesia system (Somnosuite, Kent Scientific, Torrington, CT, USA) and maintained deep anesthesia at 2.8–3.2% isoflurane at the same flow rate. After confirming a deep anesthesia level by a negative pedal pain reflex, the animals were transcardially perfused using 30 mL of ice-cold NMDG-aCSF (Solution 1). The animals were then sacrificed by decapitation. The brain was rapidly removed and placed in ice-cold NMDG-aCSF (Solution 1). The cerebellum was separated and the cerebrum was cut into four parts (sagittal along the hemispheric cleft and horizontally). 350 µm slices were prepared from each block using a McIlwain tissue chopper (The Mickle Laboratory Engineering Company Ltd., Surrey, UK). Care was taken that the process of brain extraction, from decapitation till the brain was in the ice-cold aCSF, lasted less than 2 min and the slicing procedure was done in less than 8 min. After cutting, the slices were transferred to 32–34 °C warm NMDG-aCSF (Solution 1) for 12 min for protective recovery. Then, the slices were transferred to HEPES-holding aCSF (Solution 2) at ~ 32 °C for 15–30 min incubation before transfer to the NMR spectrometer, where the slices were perfused with the aCSF of Solution 3. The second batch of cerebrum slices was kept in the HEPES-holding aCSF (Solution 2) at room temperature (25 °C) for 3–5 h prior to transfer to the NMR spectrometer.

### DNP spin polarization and dissolution

Spin polarization and fast dissolution were performed on a dissolution-DNP spin polarizer (HyperSense, Oxford Instruments Molecular Biotools, Oxford, UK) operating at 3.35 T. Microwave frequency of 94.110 or 94.114 GHz was applied for the polarization of a [1-^13^C]pyruvic acid formulation at 1.5 K. The formulations included 11.1 to 14.0 mM OX063 radical. The amount of [1-^13^C]pyruvic acid formulation placed in the polarization cup was 15 ± 0.5 mg which was dissolved in 4 mL of dissolution buffer (Solution 4). The dissolution was pressure ejected into a conical tube, which contained 6 mL of Solution 4 mixed with 2 mL of Solution 5. The waiting solution in the conical tube was bubbled with 95%/5% O_2_/CO_2_ and pre-warmed in a 40 °C water bath (Solution 6).

### NMR Spectroscopy

^31^P and ^13^C NMR spectroscopy were performed in a 5.8 T high resolution NMR spectrometer (RS2D, Mundolsheim, France) using a 10 mm broad-band NMR probe. Homogeneity optimization (shim) was performed using the water signal on the ^1^H channel and using the lock system. To support the latter, the aCSF was supplemented with 10% D_2_O in all of the experiments. ^31^P spectra were acquired with 1,640 scans over a total scan time of 30 min with a repetition time of 1.1 s and a flip angle of 50°. ^13^C spectra of metabolites in a hyperpolarized state were acquired using product selective saturating-excitation pulses^8^, applying 2.5 ms cardinal sine (sinc) pulses specifically designed to excite hyperpolarized [1-^13^C]lactate and hyperpolarized [^13^C]bicarbonate at a 90° nutation angle. Selective excitation for [1-^13^C]lactate and [^13^C]bicarbonate was done consecutively, with 4 s intervals, resulting in an 8 s interval for each metabolite. That is, we measured the accumulated production for both hyperpolarized [1-^13^C]lactate and [^13^C]bicarbonate within 8 s. For [1-^13^C]lactate detection the pulse was centered on the [1-^13^C]pyruvate hydrate frequency (179.4 ppm). For [^13^C]bicarbonate acquisition, the pulse was centered 214 Hz down-field of the [^13^C]bicarbonate frequency (161 ppm). The same acquisition strategy was previously applied for studies on other perfused tissue slices^5–7^. Hyperpolarized [1-^13^C]pyruvate experienced a flip angle of 1.07° and 4.5° in the respective selective excitations of [1-^13^C]lactate and [^13^C]bicarbonate acquisitions. The following parameters were used for the selective RF pulses: sinc shape, 2.5 ms pulse width, 24% amplitude (an RS2D system specific parameter), 45 dB attenuation power, 10.884 kHz offset for [1-^13^C]lactate excitation, and 9.063 kHz offset [^13^C]bicarbonate excitation. The response profile of this RF pulse can be found in the Supporting Information of Adler-Levi et al*.*^7^.

### Experimental workflow and setup

The NMR experiments in cerebrum slices were carried out as previously described^4^ with a few modifications. The cerebrum slices from the whole cerebrum were pooled together and then divided into two approximately evenly sized batches. While the first batch (Batch 1) was taken to the NMR spectrometer after 15–30 min of recovery in HEPES-holding aCSF (Solution 2), the second batch (Batch 2) was stored in the HEPES-holding aCSF for 3–5 h until the NMR experiment. We adopted this scheme because we found that the volume of the slices resulting from an entire rat cerebrum exceeded the volume visible by the NMR probe often by two-fold.

### Experimental workflow and setup

Cerebrum slices were continuously perfused with aCSF (Solution 3) at a flow rate of 4 mL/min. Two hundred mL of this aCSF solution were cycled between a reservoir bottle immersed in a 40 °C water bath and the NMR tube. The reservoir was continuously bubbled with humidified 95% O_2_/5% CO_2_. Inflow and outflow into/from the NMR tube was delivered via medical grade extension tubes and pumped in a closed circle with a peristaltic pump (Masterflex L/S Analog Pump Systems, Cole-Parmer, IL, USA). The inflow and outflow lines were connected to thin polyether ether ketone (PEEK) lines (inner diameter 0.040″, Upchurch Scientific, Inc., Oak Harbor, WA, USA). The magnetic susceptibility of PEEK is similar to water and therefore can be used during NMR spectroscopy recordings. The inflow line was located directly above a PEEK spacer, which ensured that the cerebrum slices were held in the sensitive zone of the NMR probe (Fig. 1). We also fixed an NMR compatible temperature probe (Osensa, BC, Canada) at the center of the probe to monitor temperature changes. To prevent slices from floating to the upper end of the NMR tube, we attached a small cotton patch approximately 8 cm above the active zone to the inflow line. Two out-flow lines, a main and a backup, spaced approximately 10 and 12 cm above the bottom of the probe delivered the out-flow at higher flow rates than the inflow to avoid overflow of the tube inside the spectrometer (Fig. 1). The temperature in the NMR tube inside the spectrometer was calibrated to 34–36 °C using the spectrometer’s temperature regulator (hot air flow).

The administration of hyperpolarized media to the cerebrum slices was done using a bypass constructed of medical grade tubing and 3-way valves as previously described^7^, to obtain constant perfusion with oxygenated hyperpolarized media with solute concentrations as close as possible to Solution 3, with the exception of 14 mM of hyperpolarized [1-^13^C]pyruvate. During regular aCSF perfusion (*i.e.* the time in between injections), the bypass was shunted by 3-way valves controlling its in- and out-flows. For injections, the hyperpolarized dissolution medium (Solution 4 with hyperpolarized [1-^13^C]pyruvate) was mixed with the concentrated aCSF solution (Solution 5) which was pre-saturated with humidified 95% O_2_/5% CO_2_ in a conical tube. The mixture was pressure-injected into the bypass tube using a manifold, thereby ejecting the fluids stored therein into a bin. The length of the bypass tube was adjusted to allow intake of 10 mL of this mixture. That is, 2 mL of this solution were ejected into a bin to ensure that the solution, which would be pumped into the NMR tube, was freshly oxygenated hyperpolarized medium with appropriate solute concentrations. After pressure-filling of the bypass with hyperpolarized medium, the 3-way valves controlling in-flow from the perfusion system to the bypass and out-flow from the bypass into the NMR tube were opened. Now, the peristaltic pump was able to push the hyperpolarized medium out of the bypass and into the NMR tube containing the cerebrum slices at a constant flow rate of 4 mL/min. We note that this constant perfusion system and the administration of the hyperpolarized medium using constant perfusion eliminated the slice motion that was associated with bolus injections described previously^4^.

As summarized in Table 1, we performed 11 paired injections (*i.e.* 11 first and 11 s injections, in total 22 injections) of hyperpolarized [1-^13^C]pyruvate in batches obtained from 7 different rats. Five of these paired injections were performed on Batch 1 and six were performed on Batch 2. Each sample contained slices from one animal’s cerebrum: A first injection was performed after ~ 45 min of constant aCSF perfusion (n = 11), and a second injection after ~ 45 min of incubation with either TTX (1 µM, n = 3), OLI (20 µg/ml, n = 3), or regular aCSF (n = 5). ^31^P NMR spectra were recorded prior to each injection of hyperpolarized material to confirm the viability of the slices by the presence of phosphocreatine and ATP signals. ^13^C spectra were recorded during the administration of the hyperpolarized medium to the slices as described above.

**Table 1:** Detailed description of hyperpolarized [1-^13^C]pyruvate injections and the experimental groups.

Animal no.*	Batch 1**		Batch 2**	
	Injection 1	Injection 2	Injection 1	Injection 2
1	NA	NA	aCSF	OLI (OLI1)
2	aCSF	TTX (TTX1)	aCSF	OLI (OLI2)
3	aCSF	TTX (TTX2)	aCSF	OLI (OLI3)
4	NA	NA	aCSF	TTX (TTX3)
5	aCSF	aCSF (CTL1)	aCSF	aCSF (CTL2)
6	aCSF	aCSF (CTL3)	NA	NA
7	aCSF	aCSF (CTL4)	aCSF	aCSF (CTL5)

*The animal number corresponds to the chronological order of the experiments.

**Batch 1 and Batch 2 were obtained from the same animal. Batch 2 was investigated after Batch 1.

NA: Data excluded due to technical difficulties during the experiment. aCSF, no pharmacological challenge. TTX, cerebrum slices incubated with tetrodotoxin; OLI, cerebrum slices incubated with oligomycin. The identifiers TTX1-3, OLI1-3, and CTL1-5 correspond with the data presented in Tables S1 and S2 in the supporting information.

### Data analysis and statistics

Spectral processing was performed using MNova (Mestrelab Research, Santiago de Compostela, Spain). Integrated intensities were calculated with MNova.

The significance of the differences between injections (*i.e.* before and after treatment) were tested using a paired Student’s t-test.

### Apparent metabolic rates and total production calculation

As we have used product selective saturating-excitation pulses^8^, the hyperpolarized metabolites were fully sampled (and depolarized) by each selective pulse, and only newly synthetized hyperpolarized metabolites were detected on the next excitation. The observed signal was equivalent to the accumulation of the specific metabolite in between two consecutive pulses^5–8^ (8 s). To quantify the corresponding metabolite production level, the [1-^13^C]pyruvate signal was used as a reference. To ensure appropriate referencing, we selected a temporal window at which the [1-^13^C]pyruvate concentration was constant (and known). In this way, the production of [1-^13^C]lactate and [^13^C]bicarbonate could be expressed in nmol/s as previously described^5–7^ and explained in detail below. The [1-^13^C]pyruvate concentration in the NMR tube first increased during the wash-in phase, then reached a plateau at a maximal concentration of 14 mM for at least 1 min, then decreased– during the wash-out phase. To be able to determine the temporal window at which the [1-^13^C]pyruvate concentration was maximal and constant (plateau), the [1-^13^C]pyruvate signal was corrected for signal decay due to T_1_ relaxation and RF pulsation, using an effective relaxation constant, T_eff_. T_eff_ was determined to be 58 s according to its ability to correct the [1-^13^C]pyruvate decay curve to display these flow characteristics (wash-in, plateau and wash-out). The corrected [1-^13^C]pyruvate signal, ${\mathrm{S}}_{\mathrm{Pyr}\_\mathrm{corr}}(\mathrm{t})$, was calculated using Eq. 1,

1 $${\mathrm{S}}_{\mathrm{Pyr}\_\mathrm{corr}}(\mathrm{t})= \frac{{\mathrm{S}}_{\mathrm{Pyr}}(\mathrm{t})}{{\mathrm{e}}^{(-\frac{\mathrm{t}}{{\mathrm{T}}_{\mathrm{eff}}})}}$$

where ${\mathrm{S}}_{\mathrm{Pyr}}(\mathrm{t})$ is the measured [1-^13^C]pyruvate signal. Only the time points within the plateau phase of the corrected [1-^13^C]pyruvate signal were used for apparent metabolic production rate determinations. These time points were selected using a criterion of no more than 10% deviation from the first maximum value of the ${\mathrm{S}}_{\mathrm{Pyr}\_\mathrm{corr}}(\mathrm{t})$ curve, as previously described^7^.

The apparent metabolite production rate was calculated using Eq. 2,

2 $${\upupsilon }_{\mathrm{product}}(\mathrm{t})= \left[\mathrm{Pyr}\right]\times \frac{{\mathrm{V}}_{m}}{\mathrm{TR}}\times \frac{{\mathrm{S}}_{\mathrm{Pro}}(\mathrm{t})}{{\mathrm{S}}_{\mathrm{pyr}}(\mathrm{t})}\times (\frac{\mathrm{sin}(\mathrm{\theta pyr})}{\mathrm{sin}(\mathrm{\theta pro})})$$

where ${\upupsilon }_{\mathrm{product}}(\mathrm{t})$ is the apparent production rate of [1-^13^C]lactate or [^13^C]bicarbonate at each time point $, \left[\mathrm{Pyr}\right]$ is the maximal [1-^13^C]pyruvate concentration that could be delivered, which is known to be 14 mM, ${\mathrm{V}}_{m}$ is the volume occupied by the medium in the NMR probe sensitive region (estimated to be 0.5 mL), TR is the excitation interval for each metabolite which is 8 s for both [1-^13^C]lactate and [^13^C]bicarbonate, θpro is the flip angle of the product (90°), and θpyr is the flip angle of [1-^13^C]pyruvate (1.07° on [1-^13^C]lactate acquisitions and 4.5° on [^13^C]bicarbonate acquisitions), ${\mathrm{S}}_{\mathrm{pro}}(\mathrm{t})$ is the signal of [1-^13^C]lactate or [^13^C]bicarbonate at each time point, and ${\mathrm{S}}_{\mathrm{pyr}}\left(\mathrm{t}\right)$ is the [1-^13^C]pyruvate signal in the same spectrum. We note that in this approach, T_1_ relaxation and variations in T_1_ of the order of the relevant T_1_s (substrate and metabolites in this study), do not affect the observed metabolites formation rate^8^.

Total production was calculated point by point, *i.e.* as the sum of all ${\upupsilon }_{\mathrm{product}}(\mathrm{t})$ calculated from the individual spectra showing a discernable and quantifiable hyperpolarized metabolite signal multiplied by TR (as opposed to multiplying the average of ${\upupsilon }_{\mathrm{product}}(\mathrm{t})$ by TR).

## Results

**Figure 2:**
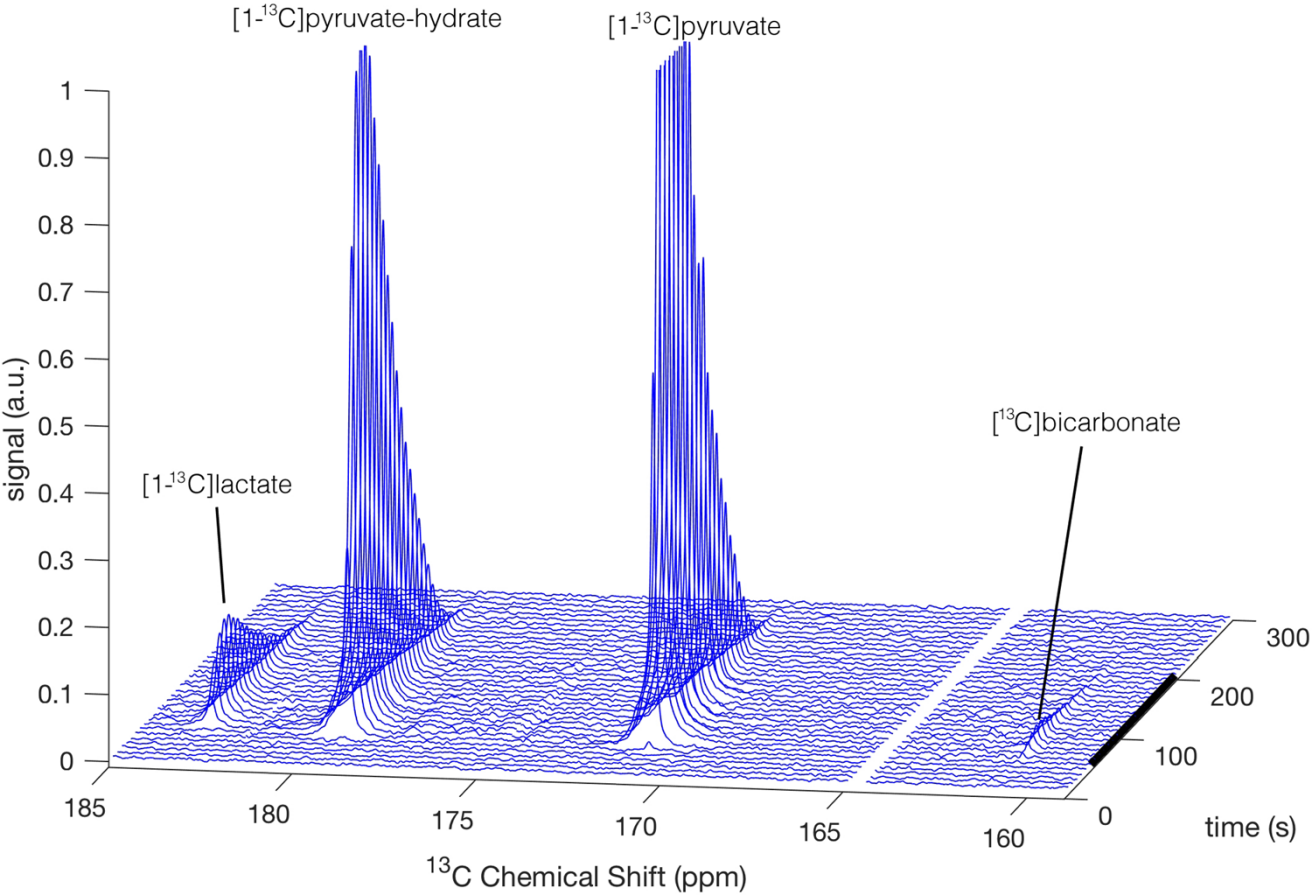
A typical example of hyperpolarized [1-^13^C]pyruvate metabolic conversion in cerebrum slices over the time course of one recording, shown as a stack plot of ^13^C NMR spectra. The [1-^13^C]lactate, [1-^13^C]pyruvate-hydrate, and [1-^13^C]pyruvate signals were obtained with one product selective pulse and the [^13^C]bicarbonate and [1-^13^C]pyruvate signals with a consecutive product selective pulse. The two pulses were consecutively applied throughout the measurement using an interleaved pulse sequence. For demonstrative purpose we combined the spectra of both interleaved pulses into one stacked 3D plot and omitted the [1-^13^C]pyruvate signal from the [^13^C]bicarbonate spectra. The [1-^13^C]pyruvate and [1-^13^C]pyruvate-hydrate signals were truncated to allow dynamic range for visibility of the [^13^C]bicarbonate signal. The black bar on the right indicates the duration of constant concentration of hyperpolarized [1-^13^C]pyruvate in the hyperpolarized media perfusing the slices.

A typical example of the measured signals and their time course is presented in Fig. 2. The temporal behavior of [1-^13^C]pyruvate metabolism was assessed by calculating the time-dependent production of [1-^13^C]lactate (LDH rate) and [^13^C]bicarbonate (PDH rate) for each acquisition. The obtained apparent rates were used for further analysis only for those time points where the concentration of hyperpolarized [1-^13^C]pyruvate was constant (and therefore known) as it was used as a reference for quantification (Methods). The duration at which each of the metabolite signals was observable within the time frame of constant hyperpolarized [1-^13^C]pyruvate concentration in the medium was determined using a threshold on the respective signal-to-noise ratio. A summation of the respective apparent rates of production over the observable duration provided the respective total production. Table 2a and 2b report on LDH and PDH activities, respectively.

**Table 2:** a LDH activity—apparent [1-^13^C]lactate production rates. b PDH activity—apparent [^13^C]bicarbonate production rates.

	Injection number	Apparent production rate (nmol/s)	Detection duration (s)	Apparent total production (nmol)
**a**				
aCSF control	1	4.82 ± 1.29	56.0 ± 6.7	301.7 ± 93.4
	2	4.12 ± 1.00	48.0 ± 4.4	219.9 ± 46.4
Tetrodotoxin (1 µM)	1	5.88 ± 1.45	56.0 ± 4.6	388.2 ± 113.3
	2	4.14 ± 0.64	77.3 ± 7.1	359.8 ± 81.6
Oligomycin (20 µg/ml)	1	3.07 ± 0.38	58.67 ± 10.67	197.7 ± 14.4
	2	4.31 ± 0.39	50.67 ± 5.33	254.5 ± 39.1
**b**				
aCSF control	1	0.41 ± 0.07	57.6 ± 5.9	26.7 ± 4.2
	2	0.26 ± 0.05	65.6 ± 4.7	19.4 ± 3.4
Tetrodotoxin (1 µM)	1	0.34 ± 0.02	69.3 ± 5.3	25.8 ± 0.7
	2	0.16 ± 0.02	29.3 ± 2.7	6.0 ± 0.6
Oligomycin (20 µg/ml)	1	0.29 ± 0.04	72.0 ± 4.6	23.2 ± 2.5
	2	0.11 ± 0.01	21.3 ± 5.3	3.4 ± 0.8

All values are given as mean ± standard error of the mean.

For TTX and OLI, Injection 1 is before incubation and injection 2 is after incubation.

**Figure 3:**
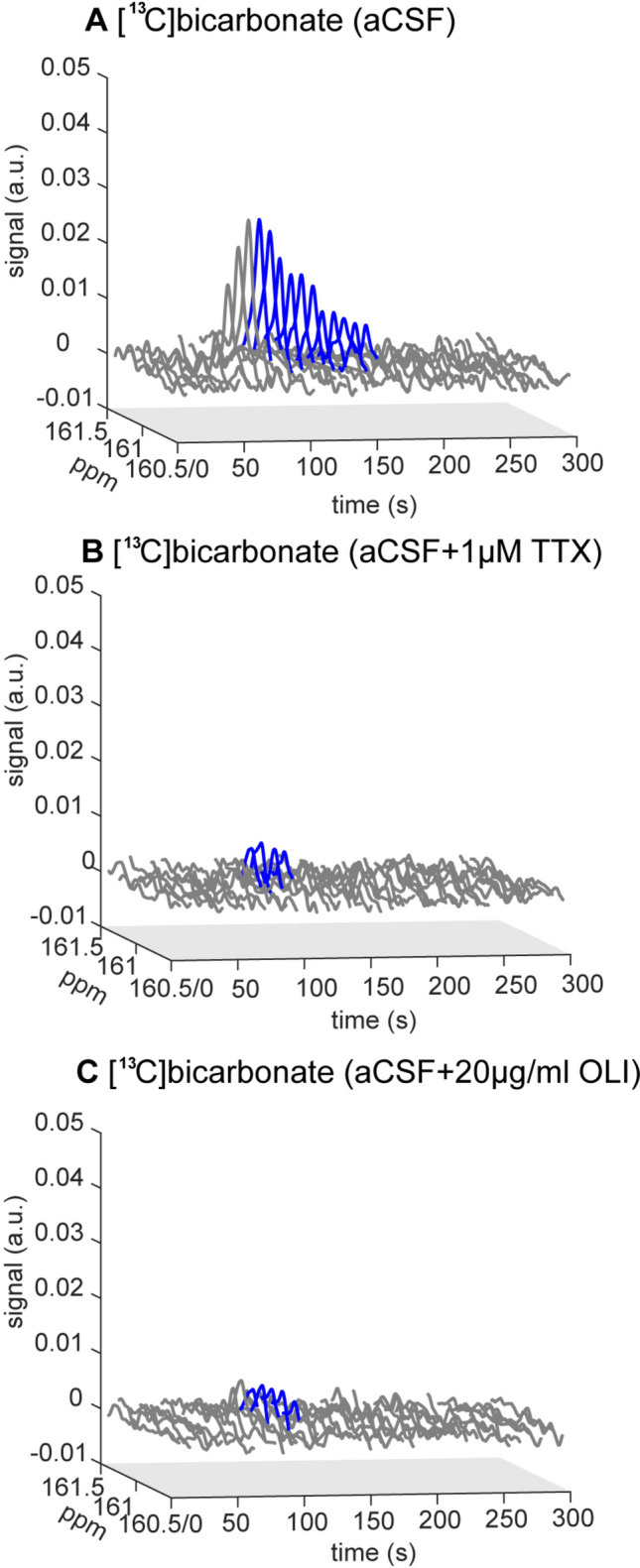
Typical hyperpolarized [^13^C]bicarbonate signal over time, acquired after incubations with aCSF (control), TTX, and OLI. (**A**) Incubation with aCSF (control). (**B**) Incubation with 1 µM TTX. (**C**) Incubation with 20 µg/ml OLI. The part of the spectrum shown in blue color is the [^13^C]bicarbonate signal used for the analysis of [^13^C]bicarbonate production (*i.e.* with sufficient SNR and under a constant [1-^13^C]pyruvate concentration). The relative intensities of these signals may be better appreciated with a collapsed time axis presented in Fig. S1 (Supporting Information).

**Figure 4:**
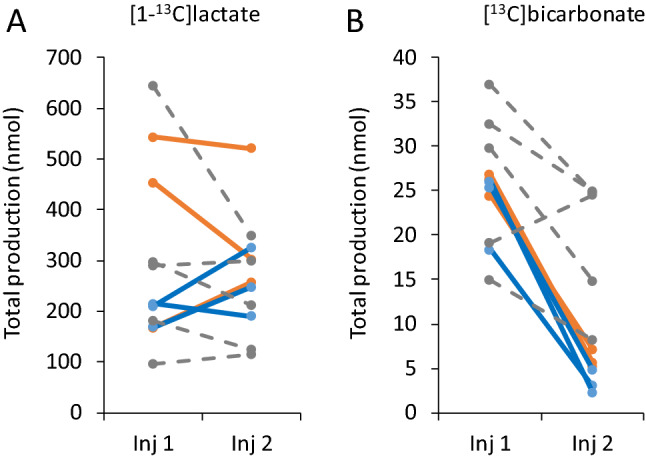
The total apparent production of [1-^13^C]lactate and [^13^C]bicarbonate prior to and following incubation with TTX, OLI, and aCSF (control). (**A**) The total production of [1-^13^C]lactate. (**B**) The total production of [^13^C]bicarbonate. TTX, tetrodotoxin, solid red lines; OLI, oligomycin, solid blue lines; Control, regular aCSF, dashed grey lines; Inj1, rates calculated on the first injection following perfusion with regular CSF; Inj2, rates calculated on the second injection, *i.e.* following perfusion with aCSF supplemented with TTX, OLI, or without supplements (Control).

This analysis showed that both TTX and OLI incubation resulted in drastic changes to PDH activity. A typical appearance of these effects is shown in Fig. 3. Figure 4B and Table 2b show that TTX incubation reduced the total [^13^C]bicarbonate production 4.4 ± 0.5 fold (n = 3), and OLI incubation reduced the total [^13^C]bicarbonate production 7.6 ± 1.9 fold (n = 3). Both induced reductions of [^13^C]bicarbonate production were significant (*p* = 0.004 and *p* = 0.015 for TTX and OLI, respectively, injection 1 and 2 are paired). Such changes were not visible in control aCSF experiments without pharmacological manipulation (a non-significant 1.5 ± 0.2 fold reduction in total [^13^C]bicarbonate production, n = 5, *p* = 0.109, injection 1 and 2 are paired). This decrease in total observed [^13^C]bicarbonate production was due to two contributing changes: 1) the time course of the [^13^C]bicarbonate signal showed a much earlier termination of detectable PDH activity after both TTX (2.4 ± 0.4 fold shorter, n = 3, *p* = 0.038) and OLI incubation (3.8 ± 0.9 fold shorter, n = 3, *p* = 0.034). Both durations significantly deviated from the durations observed in the control regular aCSF incubations (Table 2b, non-paired comparisons done on injections 2, *p* = 0.0014 for TTX *vs.* control aCSF experiments, and p = 0.00094 for OLI *vs.* contral aCSF experiments); 2) the mean apparent PDH rate averaged over these respective observation durations significantly dropped as well under TTX (2.1 ± 0.3 fold lower, n = 3, *p* = 0.033) and under OLI (2.6 ± 0.4 fold lower, n = 3, *p* = 0.043). Comparing the averaged apparent LDH rates (*i.e.* the conversion of [1-^13^C]pyruvate to [1-^13^C]lactate, Table 2a) for the three groups did not reveal statistically significant differences for the TTX group (*p* = 0.3) and the control aCSF group (*p* = 0.12), but for the OLI group a slight 1.4 fold increase was significant (*p* = 0.024). The entire dataset is provided in the Supporting Information (Note S4).

Regarding OLI incubation, an increase in [1-^13^C]lactate production was hypothesized due to shifting the cells away from ATP synthesis due to ATP-synthase inhibition. However, we did not find significant differences in the mean apparent LDH rate and apparent total production (Fig. 4A, Table 2a). In addition, the duration of the reliably detected [1-^13^C]lactate signal (sufficient SNR) did not show significant differences either.

## Discussion

We show that the shutdown of action potential generation induced by 45 min incubation with 1 µM TTX diminishes the ability to produce bicarbonate form exogenous pyruvate. Furthermore, a similar change was also evident after incubation with the antibiotic OLI. Nevertheless, we show that the activity of LDH is not affected under both conditions. The production of lactate from pyruvate relies on pyruvate transport into the cell and activity of the LDH enzyme. However, the production of CO_2_ (followed by conversion to bicarbonate) requires an additional transport process, across the mitochondrial inner membrane^23^ and the activity of PDH. Because LDH activity (as determined by hyperpolarized [1-^13^C]lactate production) was not modified by both conditions it appears that the transport of pyruvate across the plasma membrane and the activity of LDH in the cytosol were not affected by these two conditions. These results may suggest that 1) with regards to OLI incubation, LDH activity is not dependent on ATP-synthase activity for the durations investigated, *i.e.* that the NADH and NAD^+^ levels in the cytosol, which are cofactors for this reaction, were not significantly altered; and 2) with regards to TTX incubation, it appears that the reduced energy demand in the absence of action potentials did not lead to downregulation of LDH activity.

Reduced bicarbonate production could suggest that either the mitochondrial transport of pyruvate was affected or that the activity of PDH was affected. Mitochondrial pyruvate carriers facilitate the transport of pyruvate across the inner mitochondrial membrane. These carriers are symporters that use the proton gradient. Because OLI incubation will increase rather than decrease the proton gradient across the mitochondrial membrane^31^, it appears unlikely that the cause for decreased bicarbonate production under OLI incubation is reduced transport across the mitochondrial membrane. As regards to TTX incubation, we cannot speculate why such an incubation could lead to a decrease in pyruvate transport into the mitochondria.

PDH activity is regulated by pyruvate dehydrogenase kinase (PDK). Phosphorylation of PDH by PDK inactivates PDH^31^. PDK is activated by increased ratios of [ATP]/[ADP] and [NADH]/[NAD^+^]^31^. In the following we will provide potential explanation for reduced PDH activity under both OLI and TTX incubation.

OLI is an inhibitor of ATP-synthase which prevents protons from passing back into the mitochondria leading to shutdown of the proton pumps as the gradients become too high for their operation. In this way, OLI inhibits both the conversion of ADP to ATP and the transport of protons into the mitochondria^32^. NADH remains high and NAD^+^ is too low for citric acid cycle operation^31^. We suggest that the resulting high [NADH]/[NAD^+^] ratio likely activated PDK thereby leading to reduced PDH activity explaining the observation of reduced bicarbonate production activity under OLI incubation.

TTX inhibits the generation of action potentials and therefore the consumption of ATP is likely reduced. This may have led to an increase in mitochondrial ATP, and therefore the of [ATP]/[ADP] ratio. This could activate PDK and therefore reduce PDH activity, likely explaining the reduced bicarbonate production activity under TTX incubation.

Hence, we showed that the changes in pyruvate metabolism following functional manipulation of neurons are drastic and comparable to the changes induced by blockage of a vital metabolic flux. We do note that since neuronal firing is dependent on energy supply, metabolism may constrain neuronal firing^33^. Therefore, although both agents used here have different targets, we cannot exclude the possibility that a decrease in energy supply (ATP synthesis) that followed OLI incubation lead to cessation of neuronal firing and therefore in fact both conditions used here led to the loss of neural electrical activity.

Although TTX effects on metabolism have been researched already in the 1970s and 1980s^20,21^, to the best of our knowledge, its possible effect on PDH activity has not been reported. TTX has been used to modulate metabolism in several studies^22,34,35^. Nevertheless, it is still not clear whether the dominant energy substrate for neurons is glucose or lactate, and the relative contributions of glycolysis and oxidative phosphorylation remain under debate, as well their cellular origin (neurons *vs.* astrocytes)^22^. Regardless, here, TTX utilization enabled 1) differentiation between cerebrum slices with and without action potential generation ability and 2) demonstration of consequent in-cell changes in PDH activity. Such changes to in-cell enzymatic activities are not amenable for assessment in brain slices using other methodologies. Moreover, the dDNP-MR technology is translational and this sets the importance of the current finding of PDH activity as a potential in vivo non-invasive marker of brain function, subject to the limitations discussed below.

The ex vivo cerebral slices setup for NMR spectroscopy of hyperpolarized metabolites has several advantages over in vivo preclinical setups for studying the effects of neuronal shutdown on metabolism. First and foremost, the pharmacological conditions used here may be difficult to implement in vivo because they are likely to lead to permanent or transient block of nerve activity (TTX) or to acidosis (OLI). Second, a couple of technical advantages are that 1) the measured products are not contaminated with metabolites produced in the body and transported to the brain^13^; and 2) voxel contamination related to the point-spread-function that results from the acquisition/processing is avoided. This is because voxels of in vivo brain studies may contain differing amounts of both blood and brain tissue^13^ but the ex vivo preparation contains only cerebral tissue. In addition, a confounding factor concerning in vivo rodent experiments involves the blood–brain-barrier (BBB) transport kinetics via monocarboxylate transporters^36^ and their dependence on the anesthesia used^14^. Ex vivo, the BBB is severed and thus the arrival of hyperpolarized pyruvate to the cell is likely faster, as is the case for a disturbed BBB in vivo^10^.

The technical limitations of the current experimental set-up were described in detail elsewhere^4^. Briefly, our results are limited by the fact that, first, the acute cerebrum slice model arguably involves tissue damage and certainly suffers from regions of limited viability, despite our efforts to obtain healthy slices, adopting recent changes in slice preparation guidelines for mature rodents^29,30^. It is also likely that the lengthy duration of our two-phase experiments resulted in mild deterioration of slice viability over time, although this process could not be traced in a statistically significant manner as LDH and PDH activities or ATP and PCr content in control experiments (Supporting Figs. S2 and S3).

In addition, we note that our ex vivo setup does not inform about cerebral area or tissue type specific changes in metabolism, as the reported apparent rates were derived from the whole cerebrum. Hence, regional differences in TTX- or OLI-induced neuronal activity may be diluted by unaffected tissue. In another aspect, although TTX blocks sodium-channels specifically, the data do not differentiate between neuronal or glial metabolic changes following this blockage.

In addition, we note the difficulty in performing parallel biochemical / electrophysiological studies in the same slices. It is important to note that the slices perfused within the NMR tube define of the investigation system. One cannot perform classical biochemical assays or electrophysiology measurements within the NMR tube within the spectrometer. However, the effects of TTX on neural tissues are well studied^20–23^ and OLI is a veteran ATP-synthase inhibitor^31^. It may be that the confined space within the NMR tube limits the viability and health of the slices compared to a single slice that is perfused within a typical electrophysiological setup. Nevertheless, in practice this cannot be investigated in parallel.

The studies on two batches of cerebral slices taken from the same rat reduce the number of rats used per study and are in line with the guidelines for current ethical protocols. Unfortunately, due to technical problems with Batch 1 in animals 1 and 4, all of the OLI experiments were done with batch 2. Although ^31^P data and hyperpolarized [1-^13^C]lactate production data do not hint to any differences between Batch 1 and Batch 2 (*p* = 0.7, *p* = 0.3, and *p* = 0.2 for [1-^13^C]lactate production in the TTX, OLI, and control aCSF experiments, respectively), to make sure that the Batch 2 is not different from the Batch 1, it may also be useful to inspect the slices’ histopathological features after each experiment. Apoptosis/necrosis staining, and a visual inspection of neuronal cell bodies for signs of swelling or shrinkage may be informative. Unfortunatly this was not done for the current experimental groups.

Acknowledging these limitations, the results presented here could be a first indication that monitoring the metabolism of hyperpolarized [1-^13^C]pyruvate to [^13^C]bicarbonate could serve as a non-invasive reporter for inhibition of action potential generation, at very short time scales, potentially with high sensitivity. In an attempt to speculate on the relevance of the current finding to the clinical setting, the TTX group could be viewed as a model system for live but inactive (non/low firing) neural tissue. OLI was used here as a tool of metabolic intervention that is not specific for neurons. We note that previous studies have used OLI as a model for mitochondrial respiratory chain dysfunction^37^.

The potential ability to detect live but non/low firing neural tissue non-invasively could impact both basic and clinical neurological sciences. Pending successful translation of the product selective saturating-excitations acquisition approach to in vivo studies, such a technology could provide biomarkers that are directly related to electrical activity in the brain with high 3D spatial resolution. This is as opposed to fMRI which relies on the hemodynamic response that lags a few seconds behind the neural activity event and its relation to this event is complex, and with likely improved penetration and 3D spatial resolution compared to EEG measurements.

## Supplementary information

Supplementary Information.
