# Supplementary material for: Curbing action potential generation or ATP-synthase leads to a decrease in in-cell pyruvate dehydrogenase activity in rat cerebrum slices
Source: Sci Rep. 2021 May 13;11:10211. doi: 10.1038/s41598-021-89534-4 (PMC8119472; doi:10.1038/s41598-021-89534-4)
Supplement: Supplementary file 1 — Supplementary Information. [file 41598_2021_89534_MOESM1_ESM.docx]

**Curbing action potential generation or ATP-synthase leads to a decrease in in-cell pyruvate dehydrogenase activity in rat cerebrum slices**

Benjamin Grieb^1,2^, Sivaranjan Uppala^1^, Gal Sapir^1^, David Shaul^1^, J. Moshe Gomori^1^, and Rachel Katz-Brull^1*^

1 Department of Radiology, Hadassah Medical Center, Hebrew University of Jerusalem, The Faculty of Medicine, Jerusalem, Israel

2 Department of Psychiatry and Psychotherapie I (Weissenau), ZfP Suedwuerttemberg, Ulm University, Ravensburg, Germany

Supplementary information


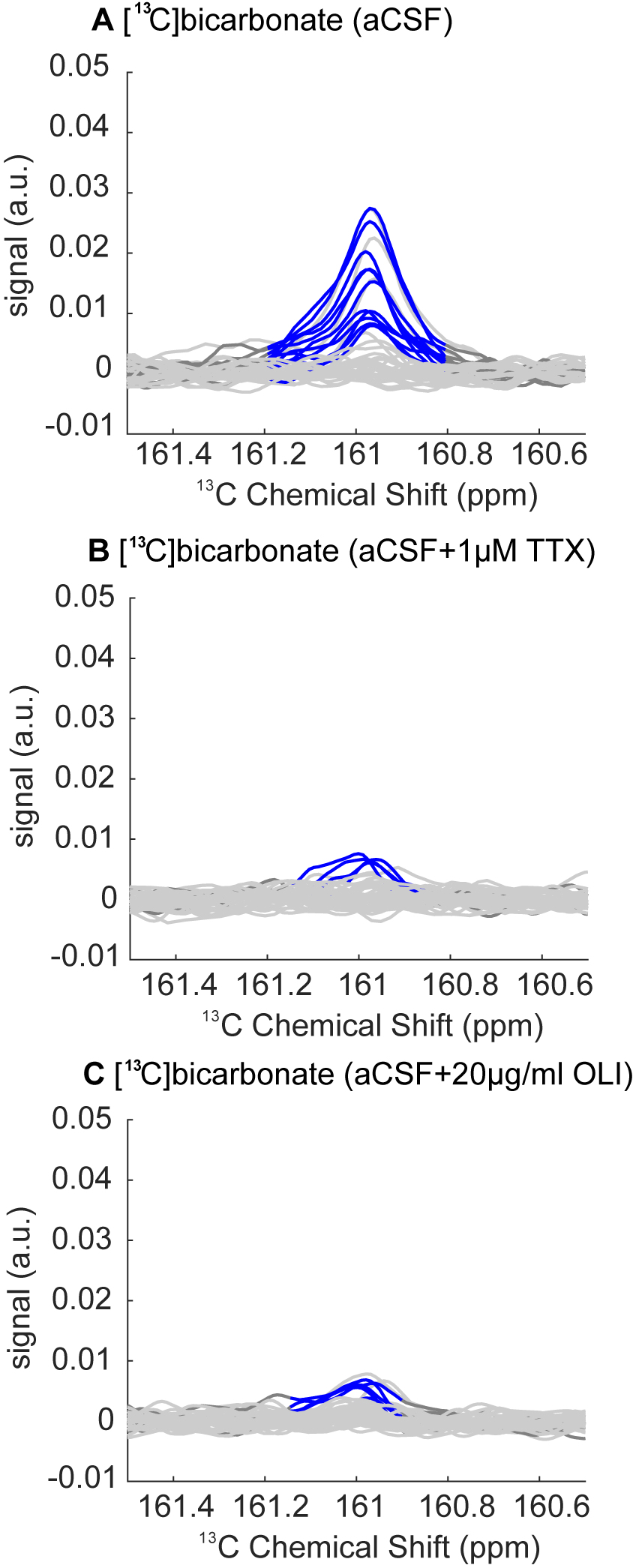


**Figure S1.**Typical hyperpolarized [^13^C]bicarbonate signals with collapsed time axis.

A) Spectra acquired after control aCSF incubation. B) Spectra acquired following1 µM TTX incubation. C) Spectra acquired following 20 µg/mL OLI incubation. The part of the spectra shown in blue color is the [^13^C]bicarbonate signal used for the analysis of [^13^C]bicarbonate production. The chemical shift range of 160.6 – 161.2 ppm was marked blue and the remaining spectrum was marked by dark grey line. Light grey lines show spectra before and after valid [^13^C]bicarbonate signal detection (not under constant [1-^13^C]pyruvate concentration). The data shown here are also shown in Figure 3 in the main text. Here, the data are presented with a 2D view collapsing the time axis.

**Typical ^31^P data and analysis of control experiments**

Figure S2 shows typical ^31^P spectra recorded from cerebral slices in control experiments. The spectra were acquired and processed as described in the Methods section and then denoised. Denoising of these thermal equilibrium ^31^P spectra was done according to Brender *et al. ^1^* using low‐rank approximation of the MR spectrum in the frequency domain*.* Briefly, the processed spectra array was treated as an m x n matrix, where m contained the spectral data, and the different time points are represented in n. This served as raw data for the denoising procedure. The raw data was then decomposed using the SVD function in Matlab (Mathworks, Natick, MA, USA), the matrix rank (r) was reduced, and then the matrix was reconstructed. We used an r of 5 for denoising the the ^31^P spectroscopy data. The reconstructed signals were then integrated, and used for further analysis. ATP and PCr content were calculated in reference to a known standard.

**Figure S2.** A ^31^P-NMR spectrum of cerebral slices used in the current study.

This spectrum was acquired from Batch 1 prior to the 1^st^ injection (see text).

PME, phosphomonoesters; Pi, Inorganic phosphate; PCr, phosphocreatine; ATP, adenosine triphosphate; ADP, adenosine diphosphate; NAD, nicotinamide adenine dinucleotide.

Figure S3. ATP and PCr levels in cerebral slices from the control group.

The data consist of 5 samples of cerebral slices from 3 animals. Inj 1 refers to the levels before the 1^st^ injection in all 5 samples (5 spectra). Inj 2 refers to the levels before the 2^nd^ injection in all 5 samples (5 spectra). Batch 1 refers to the levels prior to both injections made to Batch 1 (6 spectra). Batch 2 refers to the levels prior to both injections made in Batch 2 (4 spectra).

**Supporting Note S4. Dataset – apparent total production of hyperpolarized metabolites.**

**Table S1.** Total apparent hyperpolarized [1-^13^C]lactate production on individual injections in all experiments.

| Total [1-^13^C]lactate production (nmol) | | |
| --- | --- | --- |
|  | Inj 1 | Inj 2 |
| TTX1 | 453 | 302 |
| TTX2 | 544 | 521 |
| TTX3 | 168 | 257 |
| OLI1 | 215 | 190 |
| OLI2 | 209 | 325 |
| OLI3 | 169 | 248 |
| CTL1 | 644 | 349 |
| CTL2 | 181 | 125 |
| CTL3 | 290 | 300 |
| CTL4 | 96 | 115 |
| CTL5 | 297 | 211 |

**Table S2.** Total apparent hyperpolarized [^13^C]bicarbonate production on individual injections in all experiments.

| Total [^13^C]bicarbonate production (nmol) | | |
| --- | --- | --- |
|  | Inj 1 | Inj 2 |
| TTX1 | 26.3 | 5.2 |
| TTX2 | 24.4 | 7.2 |
| TTX3 | 26.8 | 5.7 |
| OLI1 | 18.3 | 3.0 |
| OLI2 | 26.0 | 2.3 |
| OLI3 | 25.3 | 4.9 |
| CTL1 | 37.0 | 24.8 |
| CTL2 | 15.0 | 8.2 |
| CTL3 | 32.4 | 24.9 |
| CTL4 | 29.8 | 14.8 |
| CTL5 | 19.1 | 24.5 |

**Reference**

1. Brender JR, Kishimoto S, Merkle H, et al. Dynamic Imaging of Glucose and Lactate Metabolism by C-13-MRS without Hyperpolarization. *Sci Rep* 2019; 9: 14. Article. DOI: 10.1038/s41598-019-38981-1.
